# The impact of antenatal care, iron–folic acid supplementation and tetanus toxoid vaccination during pregnancy on child mortality in Bangladesh

**DOI:** 10.1371/journal.pone.0187090

**Published:** 2017-11-01

**Authors:** Tanvir Abir, Felix Akpojene Ogbo, Garry John Stevens, Andrew Nicolas Page, Abul Hasnat Milton, Kingsley Emwinyore Agho

**Affiliations:** 1 School of Science and Health, Western Sydney University, Penrith, New South Wales, Australia; 2 Translational Health Research Institute (THRI), School of Medicine, Western Sydney University, Penrith, New South Wales, Australia; 3 Humanitarian and Development Research Initiative (HADRI), Western Sydney University, School of Social Science and Psychology, Penrith, New South Wales, Australia; 4 Centre for Clinical Epidemiology & Biostatistics (CCEB), School of Medicine and Public Health, Faculty of Health and Medicine, The University of Newcastle, Callaghan, New South Wales, Australia; TNO, NETHERLANDS

## Abstract

**Background:**

Appropriate antenatal care (ANC) is an important preventive public health intervention to ensure women’s and newborn health outcomes. The study aimed to investigate the impact of ANC, iron–folic acid (IFA) supplementation and tetanus toxoid (TT) vaccination during pregnancy on child mortality in Bangladesh.

**Method:**

A cross-sectional study of three datasets from the Bangladesh Demographic and Health Surveys for the years 2004, 2007 and 2011 were pooled and used for the analyses. A total weighted sample of 16,721 maternal responses (5,364 for 2004; 4,872 for 2007 and 6,485 for 2011) was used. Multivariate logistic models that adjusted for cluster and sampling weights were used to examine the impact of ANC, IFA supplementation and TT vaccination during pregnancy on the death of a child aged 0–28 days (neonatal), 1–11 months (post-neonatal) and 12–59 months (child).

**Results:**

Multivariable analyses revealed that the odds of postnatal and under-5 mortality was lower in mothers who had ANC [Odds Ratio (OR) = 0.60, 95% confidence interval (95% CI): 0.43–0.85], IFA supplementation [OR = 0.66, 95% CI: (0.45–0.98)] and ≥2 TT vaccinations (OR = 0.43, 95% CI: 0.49–0.78) for post-natal mortality; and for under-5 mortality, any form of ANC (OR = 0.69, 95% CI: 0.51–0.93), IFA supplementation (OR = 0.67, 95% CI: 0.48–0.94) and ≥2 TT vaccinations (OR = 0.50, 95% CI: 0.36–0.69). When combined, TT vaccination with IFA supplementation, and TT vaccination without IFA supplementation were protective across all groups.

**Conclusion:**

The study found that ANC, IFA supplementation, and TT vaccination during pregnancy reduced the likelihood of child mortality in Bangladesh. The findings suggest that considerable gains in improving child survival could be achieved through ensuring universal coverage of ANC, promoting TT vaccination during pregnancy and IFA supplementation among pregnant women in Bangladesh.

## Introduction

Child mortality is an important public health issue worldwide, and often reflects a country’s developmental status because it serves as a good indicator for antenatal care, delivery and early postnatal care as well as the health status of children [1]. Globally, the risk of child mortality is highest during the early neonatal period (the first six days of life), accounting for 15% of all under-five mortality (U5M), highlighting the significance of appropriate antenatal care, safe delivery practices and effective postnatal care. In 2015, approximately 2.6 million neonatal deaths were reported, despite a 42% decrease since 1990 [1]. Global policy attention and scale up efforts such as the Millennium Development Goals (MDGs) that focused on key aspects of improving child survival and health have played a major role in the reduction of childhood-related diseases (including diarrhoea, tetanus and measles), with subsequent impact on child mortality [1, 2]. Despite this improvement, child mortality remains a major health issue in resource-constraint communities in sub-Saharan Africa and South Asia countries, including Bangladesh [1].

Notably, most of the neonatal deaths are due to preventable diseases (such as infections and intrapartum-related complications) [1] in which appropriate access to basic and affordable interventions such as perinatal care could play a major role [3]. Studies have shown that mothers can increase the survival and health of their babies by attending antenatal care (ANC), being immunised against tetanus, and being provided with iron-folic acid (IFA) supplementation and health promotion messages such as exclusive breastfeeding [2, 4, 5].

In Bangladesh, small-scale studies have investigated the efficacy of ANC attendance and IFA supplementation in the prevention of U5M. For example, West et al. revealed that multiple antenatal micronutrients and IFA supplementation did not decrease all-cause infant mortality, but reduced the prevalence of stillbirths and preterm births [6]. In addition, Pervin et al. reported that ANC was associated with improved perinatal survival in a regional area of Bangladesh [7]. The limitation of these studies is that they were conducted in regional areas of Bangladesh, and the findings may be limited in informing country-wide interventions at the national level. A recent population-based study reported improvement in the utilisation of appropriate ANC (more than four visits) in Bangladesh between 1994 and 2014, with associated inequities across the socio-demographic scale and regional areas of Bangladesh [8]. The study did not examine the impact of ANC, IFA supplementation and tetanus toxoid (TT) vaccination during pregnancy on child mortality, which is the focus of the current study.

Maternal TT vaccination during pregnancy was incorporated into the World Health Organisation’s Expanded Program on Immunization (EPI) in the mid-1970s to prevent child mortality from tetanus and has become a standard approach to ANC [9]. Although the impact of ANC TT vaccination on child mortality has been documented [9–11], a recent systematic review indicated that only a few studies provided supporting evidence for the initiative, reflecting the dearth of data in many developing countries [9]. The paucity of evidence base for ANC TT vaccination and child mortality warrants investigation in a resource-poor country such as Bangladesh to continue advocacy for initiatives to improve child survival and extend the longevity of life.

This study aimed to investigate the impact of ANC, IFA supplementation and TT vaccination during pregnancy on child mortality in Bangladesh, using the Bangladesh Demographic and Health Survey Data (BDHS, 2004–2011). Evidence from this study will inform stakeholders in Bangladesh to advocate and intensify efforts for preventive health programmes to ensure improvement in child health outcomes.

## Methods

### Data sources

Data sets from the BDHS for the years 2004, 2007 and 2011 were pooled and used for the analyses. [12–14]. The BDHS collects socio-demographic characteristics, maternal and child health information from a nation-wide representative sample of households. The data were collected by the National Institute of Population Research and Training (NIPRT), with technical support from Inner City Fund (ICF) International and Measure DHS, using a two-stage sampling method and standardised household questionnaires. Maternal and child health information were obtained from eligible women aged 15–49 years in each household surveyed. A total weighted sample of 16,721 maternal responses (5,364 for 2004; 4,872 for 2007 and 6,485 for 2011) was used for this study, with an average response rate of 98%. The 2014 BDHS datasets were not incorporated into the analyses because information on TT vaccination during pregnancy was not collected from respondents. Additional information on the BDHS methodological strategy for data collection has been described in detail elsewhere [12–14].

### Study outcome

The outcome variable for the study was child mortality, which comprises neonatal (death within 0–28 days), post-neonatal (death from 1 to 11 months) and child (death between 12 and 59 months) mortalities.

### Main exposure variables

The main variables for the study comprise ANC, IFA supplementation and TT vaccination during pregnancy. ANC services may include offering maternal health education, physical examination of the pregnant woman, IFA supplementation and TT vaccination. The impact of each exposure variable and different combinations of ANC, IFA supplementation and TT vaccination on child mortality was investigated in the study.

### Study factors

The study adapted the Mosley and Chen model for determinants of child mortality [15]. We identified four main variables in the current study: (i) Community-level factors [place of residence (rural/urban) and administrative region]; (ii) Socio-economic determinants (maternal marital status, maternal religion, maternal age at the time of the survey, maternal age at the time of birth of the child, parents’ working status, maternal body mass index (BMI), parents’ level of education, household wealth index, maternal access to the mass media: radio, television and newspaper/magazine); (iii) Proximate determinants (gender of baby, birth rank and interval, maternal desire for pregnancy); and (iv) Pregnancy or delivery at health-care service, use of ANC services, including IFA supplementation, use of TT during pregnancy; place and mode of delivery of baby and type of delivery assistance received.

All households across the three surveys were ranked by using the household wealth index, which was constructed by NIPRT and Measure DHS by apportioning weights to three characteristics of households. These included: the type of floor and wall; access to electricity; and six household assets; namely, possession of radio, television, bicycle, motorcycle, car and fridge.

### Statistical analysis

Frequency tabulations were used to describe characteristics of the study population. Logistic regression models were used to perform analysis to determine the unadjusted and adjusted odds ratios (ORs) for determinants of neonatal, post-neonatal and U5M. In addition, the independent effect of each variable after other covariates were controlled for, was investigated in multivariate analyses.

The modelling was performed in 2 stages. Firstly, a significance level of 0.05 was used to remove all factors not significant among the various variables (community-level and socio-economic). This was done by performing a backward stepwise elimination procedure. Regardless of their level of significance, the year of the survey and maternal age at birth of the child were retained in the final model [16]. Secondly, ANC, mode of delivery, place of delivery and type of skilled delivery assistance were assessed, after controlling for significant community and socio-economic variables. The type of skilled delivery assistance has been known to have a protective effect on child mortality [17]. Consequently, this variable was retained in the final model, regardless, other non-significant variables were removed. ANC was found to be correlated with both IFA supplementation and TT vaccination. Hence, the study then examined the effect of a combination of any form of ANC and IFA supplementation, as well as a combination of IFA and TT vaccination on neonatal, post-neonatal and under-5 mortality.

The ORs and corresponding 95% confidence interval for neonatal, post-neonatal and U5M, and each study factor were examined and reported. The statistical software package Stata version 12 (Stata Corporation, College Station, USA) was used for all statistical analyses. The ‘svy’ command was used to adjust for sampling weights and the cluster survey design.

### Ethics approval

The study was based on the analyses of existing survey datasets that are available to apply for online, with all identifier information removed. The surveys were approved by the Ethics Committee of the ICF International, USA and the National Research Ethics Committee of Bangladesh Medical Research Council (BMRC), Bangladesh. We obtained approval from Measure DHS to download and use the data for this study.

## Results

### Characteristics of the study population

The study was based on a total weighted sample of 16,722 eligible women. As shown in Table 1, there was a variation in child mortality in the administrative regions of Bangladesh, with the lowest and highest mortality occurring in the Barisal (6.4%) and Dhaka (32.9%) regions, respectively. Less than 1 in 5 families had both parents in paid employment in the 12 months preceding the survey, and nearly 50% of mothers had no schooling and were from poor households. More than three-quarters of mothers gave birth at home, and nearly one-third delivered their babies with assistance from traditional birth attendants.

**Table 1:** Characteristics of the study participants (n = 16722) in Bangladesh (2004–2011).

Variable	n*	%*
**Year of survey**		
2004	5364	32.1
2007	4872	29.1
2011	6485	38.8
**Community**		
**Place of residence**		
Urban	3739	22.4
Rural	12983	77.6
**Region**		
Barisal	1067	6.4
Chittagong	3697	22.1
Dhaka	5497	32.9
Khulna	1804	10.8
Rajshahi	3377	20.2
Sylhet	1278	7.6
**Socioeconomics**		
**Maternal marital status**		
Married	16367	97.9
Formerly Married (divorced/separated/widowed)	354	2.1
**Religion**		
Islam	15382	92.0
Other	1339	8.0
**Mother's age at interview**		
15–24 years	8273	49.5
25–34 years	6772	40.5
35–49 years	1670	10.0
**Mother’s age at child's birth**		
less than 20 years	5019	30.0
20–29 years	8921	53.4
30–39 years	2560	15.3
40+ years	221	1.3
**Mother working status**		
Not working	13509	80.8
Working	3210	19.2
**Mother BMI**		
≥ 18,5	3929	23.7
18–25	11019	65.9
25+	1708	10.4
**Parents employment status**		
Father only working	13119	78.5
Both working	2998	17.9
Neither working	604	3.6
**Maternal education**		
No education	7818	46.8
Primary	7010	41.9
Secondary or above	1882	11.3
**Paternal education**		
No education	8512	50.9
primary	5331	31.9
Secondary or above	2861	17.2
**Household wealth Index**		
Rich	2921	17.5
Middle	5991	35.8
Poor	7810	46.7
**Watches television every week**		
Yes	9484	56.7
No	7234	43.3
**Listens to radio every week**		
Yes	4259	25.5
No	12458	74.5
**Read newspaper**		
Yes	2620	15.7
No	14099	84.3
**Proximate**		
**Gender**		
Female	8173	48.9
Male	8549	51.1
**Birth rank and birth interval**		
2nd/3rd birth rank, more than 2 years interval	6438	38.5
1st birth rank"	5241	31.4
2nd/3rd birth rank, less than or equal to 2 years interval	1110	6.6
4th birth rank, more than 2 years interval	3267	19.5
4th birth rank, less than or equal to 2 years interval	665	4.0
**Desire for pregnancies**		
Current	11625	69.5
Later	2598	15.6
Not at all	2497	14.9
**Pregnancy or delivery health-care service**		
**Use of antenatal care**		
Not used	6679	39.9
Used	10035	60.1
**IFA Supplements**		
No	11039	66.2
Yes	5476	32.8
**TT vaccination during pregnancy**		
Never	3573	21.4
One TT	3731	22.3
2+ TT	9385	56.3
^$^**Combined place and mode of delivery**		
Health facilities without Caesarean	1153	6.9
Health facilities with Caesarean	1482	8.9
Home	13663	81.7
^$^**Delivery assistance**		
None	195	1.2
Doctor	2561	15.3
Nurse/midwife	1054	6.3
TBA	5505	32.9
Other untrained personnel	5986	35.8

*Weighted for the sampling probability;

IFA: iron–folic acid supplementation; TT: tetanus toxoid; TBA: traditional birth attendance;

^**$**^Variables with missing data

### Factors associated with neonatal, postnatal and child mortality

Neonatal mortality was significantly associated with high maternal BMI (25kgm^-2^ or higher) compared to low maternal BMI (18.5kgm^-2^ or lower) (Table 2). Mothers with education were less likely to experience neonatal, postnatal and child deaths compared to mothers with no schooling. Postnatal mortality was significantly associated with infants of older mothers (aged ≥ 40 years) compared to those of younger mothers (aged <20 years). Employed mothers were significantly more likely to experience child mortality compared to mothers not in employment. The odds of a mother experiencing child mortality were higher among 4^th^ born infants/children where there was an interval of 2 years or less with the preceding child.

**Table 2:** Multivariate analysis of community and socio-economic factors associated with neonatal, postnatal and child mortality in Bangladesh (2004–2011).

Variable	Neonatal mortality		Postnatal mortality		Child mortality	
	AOR (95% CI)	P-value	AOR (95% CI)	P-value	AOR (95% CI)	P-value
Year of survey						
2004			1.00		1.00	
2007			0.74 (0.51,1.08)	0.120	0.71 (0.51,0.99)	0.042
2011			0.58 (0.39,0.86)	0.007	0.64 (0.45,0.90)	0.011
**Place of residence**						
Urban						
Rural						
**Region**						
Barisal					1.00	
Chittago					0.90 (0.55,1.48)	0.680
Dhaka					0.85 (0.51,1.40)	0.519
Khulna					0.37 (0.18, 0.76)	0.007
Rajshahi					0.80 (0.47,1.36)	0.419
Sylhet					1.24 (0.75, 2.03)	0.396
**Maternal marital status**						
Married			1.00		1.00	
Formerly Married			2.97 (1.57, 5.63)	0.001	3.08 (1.82,5.23)	<0.001
**Maternal education**						
No education	1.00		1.00		1.00	
Primary	0.70 (0.53, 0.91)	0.007	0.64 (0.44, 0.94)	0.023	0.75 (0.54,1.04)	0.082
Secondary or more	0.48 (0.31, 0.75)	0.001	0.19 (0.07, 0.52)	0.001	0.15 (0.06, 0.42)	<0.001
**Mother employment status**						
Not working					1.00	
Working					1.75 (1.28, 2.41)	0.001
**Mothers age at child's birth**						
Less than 20 years			1.00			
20–29 years			1.14 (0.67, 1.93)	0.632		
30–39 years			1.45 (0.75, 2.81)	0.271		
40+ years			3.03 (1.18, 7.77)	0.021		
**Mother BMI**						
≥ 18,5	1.00					
18–25	0.99 (0.76, 1.31)	0.966				
25+	1.66 (1.11, 2.46)	0.013				
**Birth rank and birth interval**						
2nd/3rd birth rank, more than 2 years interval	1.00		1.00		1.00	
1st birth rank"	1.85 (1.38, 2.48)	<0.001	1.25 (0.72, 2.15)	0.425	1.00 (0.68, 1.48)	0.980
2nd/3rd birth rank, less than or equal to 2 years interval	2.17 (1.43, 3.31)	<0.001	0.92 (0.41, 2.08)	0.843	0.97 (0.52, 1.82)	0.934
4th birth rank, more than 2 year interval	1.25 (0.89, 1.77)	0.202	1.44 (0.89, 2.33)	0.142	1.40 (0.97, 2.03)	0.071
4th birth rank, less than or equal to 2 years interval	1.99 (1.20, 3.29)	0.007	3.42 (1.98, 5.92)	<0.001	2.99 (1.88, 4.76)	<0.001

BMI: Body mass index; AOR: Adjusted Odds ratio; Only significant data shown.

The likelihood of a mother experiencing neonatal mortality was significantly higher among mothers who received no IFA supplements and had less than two TT vaccinations compared to those who had no IFA supplements but had more than two ANC TT vaccinations (Table 3). The odds of postnatal and child mortality were significantly higher among infants/children whose mothers received no IFA supplements and had less than 2 ANC TT vaccinations compared to those whose mothers received more than two ANC TT vaccinations, irrespective of whether they also received IFA supplementation. Mothers who had ANC visits and received IFA supplements were significantly less likely to experience postnatal and child deaths compared to those who had no ANC visits and received no IFA supplements.

**Table 3:** Effects of iron-folic acid supplementation, tetanus toxoid vaccination during pregnancy and antenatal care on neonatal, postnatal and child mortality in Bangladesh (2004–2011).

Variable	Number of live births	Number of deaths	Unadjusted		Adjusted	
			OR (95% CI)	P value	AOR (95% CI)	P value
***Neonatal Mortality (0–28 days)***						
**Combination of IFA supplementation and TT vaccination**						
No IFA supplements with < 2 TT injections	5492	117	1.00		1.00	
No IFA supplements with ≥ 2 TT injections	5725	93	0.65 (0.49, 0.86)	0.003	0.62 (0.47, 0.83)	0.001
IFA supplements with < 2 TT injections	1813	33	0.84 (0.58, 1.22)	0.364	0.77 (0.51, 1.15)	0.202
IFA supplements with ≥ 2 TT injections	3660	66	0.71 (0.52, 0.96)	0.027	0.60 (0.42, 0.86)	0.005
**Combination of ANC and IFA supplementation**						
No ANC and no IFA supplements	5663	114	1.00		1.00	
ANC without IFA supplements	5577	96	0.85 (0.65, 1.12)	0.257	0.98 (0.73, 1.32)	0.908
IFA supplements alone	1015	22	0.96 (0.59, 1.57)	0.865	0.96 (0.58, 1.59)	0.879
ANC including IFA supplements	4458	78	0.82 (0.61, 1.10)	0.189	0.90 (0.65, 1.25)	0.523
***Postnatal Mortality (1–11 months)***						
**Combination of IFA supplementation and TT vaccination**						
No IFA supplements with < 2 TT injections	5422	70	1.00		1.00	
No IFA supplements with ≥ 2 TT injections	5687	39	0.56 (0.38, 0.82)	0.003	0.56 (0.38, 0.83)	0.004
IFA supplements with < 2 TT injections	1791	22	0.83 (0.51, 1.36)	0.469	0.76 (0.45, 1.28)	0.302
IFA supplements with ≥ 2 TT injections	3638	23	0.40 (0.25, 0.66)	<0.001	0.37 (0.22, 0.63)	<0.001
**Combination of ANC and IFA supplementation**						
No ANC and no IFA supplements	5586	78	1.00		1.00	
ANC without IFA supplements	5546	31	0.40 (0.27, 0.60)	<0.001	0.66 (0.43, 1.01)	0.057
IFA supplements alone	1002	14	0.89 (0.49, 1.60)	0.692	0.82 (0.45, 1.50)	0.516
ANC including IFA supplements	4427	31	0.40 (0.26, 0.62)	<0.001	0.51 (0.32, 0.81)	0.005
***Child Mortality (1–4 years)***						
**Combination of IFA supplementation and TT vaccination**						
No IFA supplements with < 2 TT injections	5397	95	1.00		1.00	
No IFA supplements with ≥ 2 TT injections	5669	56	0.59 (0.43, 0.82)	0.002	0.59 (0.42, 0.83)	0.002
IFA supplements with < 2 TT injections	1789	24	0.69 (0.43, 1.09)	0.111	0.66 (0.41, 1.07)	0.094
IFA supplements with ≥ 2 TT injections	3625	35	0.48 (0.32, 0.71)	<0.001	0.45 (0.29, 0.70)	<0.001
**Combination of ANC and IFA supplementation**						
No ANC and no IFA supplements	5562	101	1.00		1.00	
ANC without IFA supplements	5527	50	0.46 (0.33, 0.65)	<0.001	0.77 (0.54, 1.11)	0.161
IFA supplements alone	998	17	0.84 (0.49, 1.42)	0.512	0.79 (0.46, 1.36)	0.400
ANC including IFA supplements	4416	42	0.43 (0.30, 0.62)	<0.001	0.57 (0.38, 0.84)	0.005

IFA: Iron-folic acid supplementation; TT: tetanus toxoid; ANC: Antenatal care; AOR: Adjusted Odds ratio

**Fig 1:**
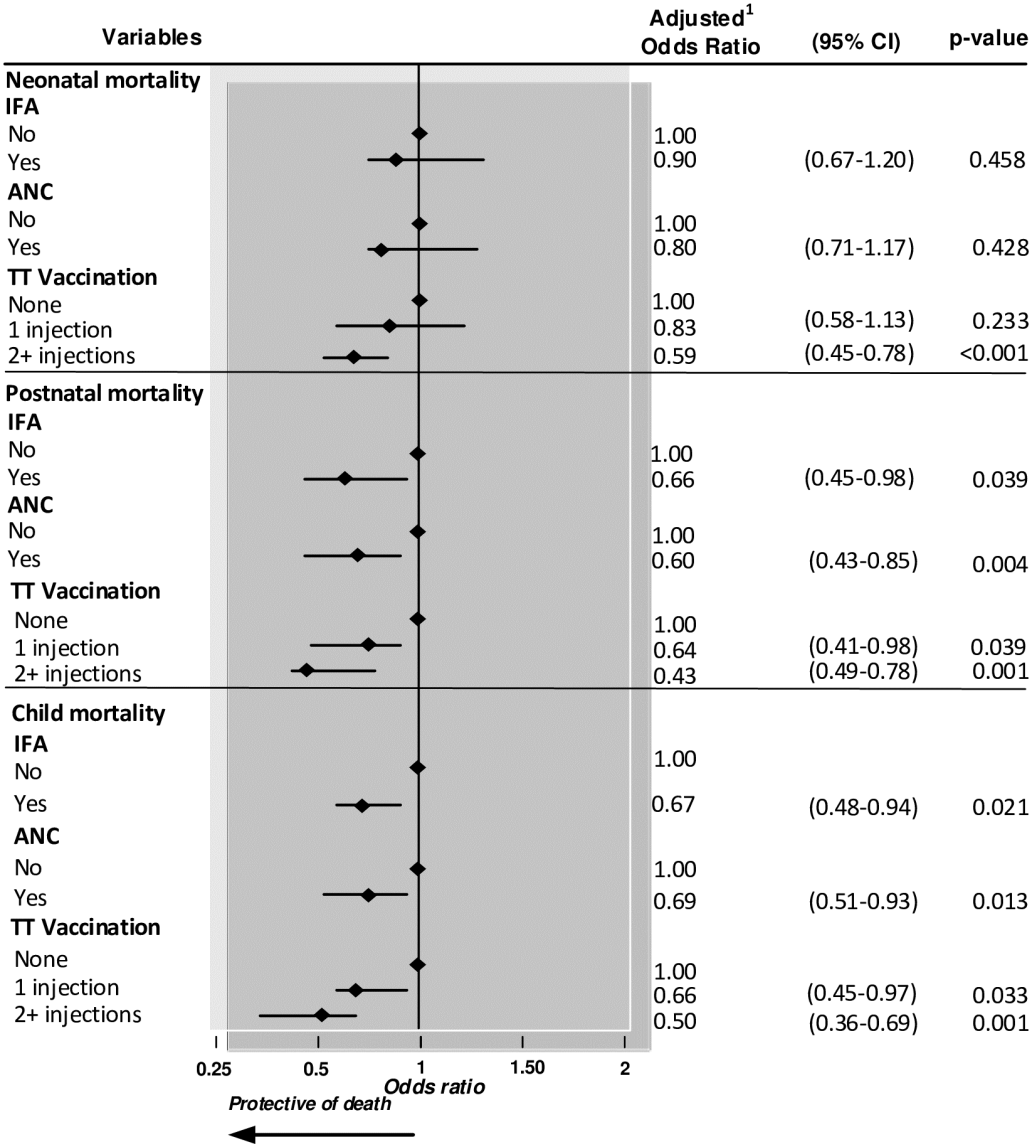
Impact of antenatal care, iron-folic acid supplementation and tetanus toxoid vaccination on child mortality in Bangladesh (2004–2011). Adjusted for place of residence, region; maternal marital status, mother's age at interview, mothers age at child's birth, mother working status, mother BMI, parents employment status; maternal highest level of education; paternal highest level of education; household wealth Index; watches television every week; listens to radio every week; read newspaper; sex of the baby; previous birth rank and birth interval, and desire for pregnancies. TT—tetanus toxoid; IFA—Iron-folic acid supplementation; ANC—antenatal care.

Neonates whose mothers received no ANC TT vaccinations were significantly more likely to die compared to those whose mothers had more than two ANC TT vaccinations (Fig 1). The likelihood of postnatal and child mortality was significantly higher among infants/children whose mothers attended no ANC clinics, did not receive IFA supplements and TT vaccinations during pregnancy.

## Discussion

In the current study, we found that the provision of any form of ANC intervention led to a reduction of the likelihood of postnatal and child mortality (but not neonatal mortality) in Bangladesh. In particular, IFA supplementation and TT vaccinations during pregnancy, two key components of ANC were found to be protective against infant/child mortality, reflecting the importance of ANC in improving maternal and child health outcomes.

Our study showed that there was a decline in child mortality between 2004 and 2011, consistent with findings from the GBD study 2015 [1] and United Nations MDG 2015 report [18]. The improvement in child health may be due to a number of global and national initiatives, including the WHO EPI [19], MDGs, national government policies and involvement of non-governmental organisations [20, 21]. We also found that IFA supplementation had a protective effect against neonatal, postnatal and child mortality. This finding is consistent with results of a reported trial of micronutrient supplementation during pregnancy in rural China in which neonatal mortality was reduced by 54% in mothers who received regular IFA supplementation compared to mothers who received only folic acid [22]. The benefits of IFA supplementation for neonatal mortality and pregnant women were highlighted in a study from Indonesia, [23] and other developing countries [24], respectively. These findings suggest that the provision of IFA supplementation to pregnant women should be promoted and supported at all levels of the healthcare system in Bangladesh. Efforts are being made by many organisations in Bangladesh to encourage routine use of IFA supplementation for pregnant women. For example, the Micronutrient Initiative (MI) in Bangladesh is one of the non-governmental organisation which strives to increase the number of pregnant women receiving appropriate IFA supplements [25]. A key aspect of the MI is to ensure that pregnant women in hard-to-reach areas receive micronutrient supplements to improve women and newborn survival and health.

In the study, we found a protective effect against neonatal, postnatal and child mortality from ANC TT vaccination (two or more vaccinations) compared to IFA supplementation alone, consistent with published reports [26–28]. This finding suggests that pregnant women should, therefore, be encouraged to take TT vaccinations in combination with IFA supplementation, which is part of the standard approach to ANC. In Bangladesh, a recent report has indicated that infant mortality rate fell from 87 per 1,000 in 1990 to 43 per 1,000 live births in 2011, and U5M rate declined substantially from 151 per 1,000 to 53 per 1,000 live births over the same period. These improvements have been attributed to improved coverage of effective childhood initiatives to prevent or treat the common causes of U5M and improvements in the socioeconomic status of the Bangladeshi populations [29]. The interventions that played significant roles in reducing U5M in Bangladesh included improved coverage of childhood immunisation; effective treatment of diarrhoeal diseases and upper respiratory tract infections; improvements in breastfeeding status; and strategic implementation of integrated management of childhood illness. Other important contributing factors to reduced U5M included improved female education, increased health system strengthening and investment and strong political will to improve maternal and child health in the country [29].

Sustainable Development Goal–3.2 (SDG–3.2) aims to end preventable deaths of newborns and children under 5 years of age, with all countries aiming to reduce neonatal mortality to at least as low as 12 deaths per 1,000 live births and under-5 mortality to at least as low as 25 deaths per 1,000 live births by 2030 [30]. Although the number of under-5 mortality has decreased in Bangladesh since 1990, accelerated and sustained progress would be made by implementing timely, evidenced-based and context-specific interventions [1, 18]. Those initiatives may include increasing universal access to appropriate ANC [31]; promoting childhood immunisation [32]; access to potable water and sanitary environment; exclusive breastfeeding [33, 34] and implementation of Global strategic initiatives such as the drowning prevention intervention [21].

The study showed that higher maternal educational level was associated with a lower likelihood of neonatal, postnatal and child mortality, consistent with evidence from Nigeria [35]. The analyses also indicated that maternal employment was associated with a higher likelihood of child mortality. Detail information on the determinants of child mortality in Bangladesh has been described elsewhere [36]. In addition to ensuring that women have appropriate ANC services in Bangladesh, other key aspects of the SDGs—Goal 1, 2 and 4, which advocate for ending poverty, no hunger and improving quality education, respectively—would have a positive impact on child survival and health in Bangladesh. Furthermore, evidence has shown that people who adhere to recommended treatment or health messages have better health outcomes compared to those who do not comply with treatment or health messages [37–39]. Therefore, health, education, socio-economic and other government and non-government agencies in Bangladesh must work together towards the achievement of the SDGs and should draw on experiences and capability from the MDGs implementation to further accelerate reductions in child mortality.

The study has several strengths. First, it was based on nationally representative surveys and utilised three different Bangladesh DHS datasets, consisting of a large sample size. Second, the data analysed were restricted to information of each mother’s latest birth, five years prior to each survey. This restriction enhanced the validity of the analysis and also minimised recall bias, consistent with previous reports [40, 41]. Third, the substantial sample size made it possible to examine the effects of the main preventive interventions on mortality; notably, ANC services, IFA supplementation and TT vaccinations, and the combination of these factors, while controlling for community, socio-economic and proximate factors.

Some limitations of our study are worthy of note. First, reports from mothers could not be validated. That notwithstanding, child mortality has been a core feature in the various DHS programmes, and there has been a careful examination of methods used in the survey for many years [42]. Second, report of usage of IFA supplementation during pregnancy and other ANC services by a mother was based on her recall; consequently, we could not assess any improvement in her iron status or information on other services that the mother may have received during the antenatal visits. This may lead to under- or over-estimation of the association between the exposure variables and outcome measures. Third, there could be an underestimation of these mortalities because both the history of birth and infant/child survival data were obtained only from surviving mothers [41]. Fourth, data on the cause of death were not available, information that would have provided an opportunity for targeted and context-specific intervention. Nonetheless, detailed information on cause-specific mortality in Bangladesh has been reported in detail elsewhere [43].

## Conclusion

Our study found that a combination of IFA supplementation and ANC TT vaccination were protective against postnatal and child mortality in Bangladesh. The study also found that a minimum of two ANC TT vaccinations was protective against neonatal, postnatal and child mortality and, as a single intervention, conferred greater protection across all age groups compared to no IFA supplementation and less than two ANC TT vaccination. In Bangladesh, considerable gains could be achieved in improving child survival and extend the longevity of life through ensuring universal access to appropriate ANC, promotion of TT vaccination during pregnancy and IFA supplementation in pregnant women.
